# Porous Architecture in 3D Food Printing: Advances in Formulation, Process Control, and Sustainable Structural Design

**DOI:** 10.1111/1541-4337.70304

**Published:** 2025-10-10

**Authors:** Lorenzo Lombardi, Luigi Davide Gala, Claudio Esposito, Daniele Tammaro

**Affiliations:** ^1^ Department of Chemical, Materials and Production Engineering University of Naples Federico II Naples Italy

**Keywords:** 3D food printing, porous structure, additive manufacturing, food sustainability, porosity, hierarchical structures

## Abstract

3D food printing offers precise control over the shape, texture, and nutritional profile of edible structures, enabling high customization. A key yet under‐explored feature in this field is the internal porous architecture, which significantly affects mechanical strength, texture, and overall functionality. This review examines how ingredient formulation‐particularly polysaccharides and proteins‐and printing parameters such as temperature, flow rate, velocity, and nozzle diameter influence the formation of inter‐strand macropores and intra‐strand micropores. Emphasis is placed on pore‐generation and stabilization techniques, especially freeze‐drying and emulsion templating, which enable the creation of well‐defined porous networks. The intentional design of hierarchical porosity‐combining both macro‐ and micro‐scale structures‐emerges as a promising strategy for producing lightweight, mechanically robust, and sensorially rich food products. These porous architectures can reduce raw material consumption while maintaining consumer appeal, aligning with goals of food sustainability. In this context, porosity is not merely a structural attribute but a critical design variable for optimizing performance and environmental impact. By connecting internal structure with functional outcomes, this review aims to support the development of next‐generation 3D‐printed foods that are resource‐efficient, nutritionally tailored, and aligned with sustainable production practices.

## 1 Introduction

3D food printing is an emerging technology that adapts principles of additive manufacturing to produce edible structures with customized shapes, textures, and nutritional profiles (Yang et al. 2017). By integrating digital design with culinary techniques, it enables precise control over ingredients and allows for personalization in flavor (Liu et al. 2017), nutrition (Burke‐Shyne et al. 2021; Zhong et al. 2023), geometry (Guénard‐Lampron et al. 2021), and even visual appearance, such as color (Wen et al. 2022). This versatility makes 3D food printing valuable in a variety of fields, including gastronomy, nutrition, and sustainable food production (Sun et al. 2015).

The origins of 3D food printing can be traced back to 2006, when the first prototype printer was introduced (Malone and Lipson 2007). Early machines were limited to basic formulations such as chocolate and sugar due to constraints in extrusion technology. However, rapid advances in material handling, formulation science, and digital control have significantly expanded the capabilities of this technology (Voon et al. 2019). Modern research now spans aesthetic applications, such as personalized or artistic food designs, and functional applications aimed at improving nutrition and sustainability‐particularly through the use of alternative proteins like algae (Thakur et al. 2023; Donn et al. 2022) and insects (Severini et al. 2018). Other ongoing efforts focus on ingredient safety (Baiano 2022), product stability during and after printing (Hussain et al. 2022), and enhancing process efficiency and scalability. This broadening scope is reflected in the exponential rise in research publications over the past decade.

The printing process itself resembles conventional 3D printing, relying on layer‐by‐layer deposition of edible materials‐known as “inks”—which are typically pastes or gels derived from chocolate, dough, purees, or protein‐rich formulations. These inks must possess suitable rheological properties for extrusion and shape retention, making printability a central focus of research (Voon et al. 2019). Numerous studies investigate how ingredient type, additives, and rheological behavior influence the final printed structure (Bareen et al. 2023; Zheng et al. 2021; Liu et al. 2019b). Key printability metrics, such as shape fidelity and dimensional accuracy, are commonly used to evaluate how closely printed objects conform to their digital designs (Waseem et al. 2024).

Beyond printability and external geometry, the internal microstructure‐particularly micro‐porosity‐plays a critical role in defining the mechanical, sensory, and nutritional properties of printed foods. For instance, attributes like chewability (Shi et al. 2023; Shahbazi et al. 2021a) and digestibility (Liu et al. 2022b) are directly influenced by internal porosity. Although several studies report porosity changes in response to formulation variations (Azam et al. 2018; Liu et al. 2020c), this aspect is often secondary to the goal of optimizing shape or print resolution. As a result, the literature contains a large but fragmented body of data on porous structures in 3D‐printed foods.

Importantly, the investigation of porosity in food printing is not only relevant for enhancing texture or functionality—it also aligns with broader goals in sustainable design (Ahmadzadeh and Ubeyitogullari 2023b). In materials science, porous structures such as foams and 3D‐printed polymers are widely utilized due to their high strength‐to‐weight ratios and ability to deliver mechanical performance with reduced material use (Zheng et al. 2018; Tammaro et al. 2022a, 2025; Lombardi et al. 2025c). Applying this principle to food, the design of low‐density, micro‐porous edible structures could enable the production of satisfying, nutritionally balanced food products using fewer raw ingredients (Jarpa‐Parra and Chen 2021). This could contribute to reducing food waste and consumption, while maintaining consumer acceptance by mimicking the sensory experience of traditional, denser products. In this context, porosity becomes a key design parameter for resource‐efficient and sustainable food systems.

In addition to ingredient formulation, process parameters such as extrusion speed (Le Tohic et al. 2018), printing speed (Derossi et al. 2018), extrusion temperature (Liu et al. 2020c), and nozzle size (Montoya et al. 2021) also influence the quality of printed products. However, most studies focus on optimizing these variables for printability, often overlooking their effects on internal porosity. Consequently, a systematic understanding of how process parameters affect internal structure is still lacking.

This review aims to address that gap by organizing and synthesizing existing research on the internal porosity of 3D‐printed food products. Specifically, it explores how formulation and process variables influence the micro‐ and nano‐scale internal architecture of printed samples, with the broader goal of guiding the development of food products that are both functionally optimized and resource‐efficient.

## 2 Formation of Pores in Food‐Based Materials

**FIGURE 1:**
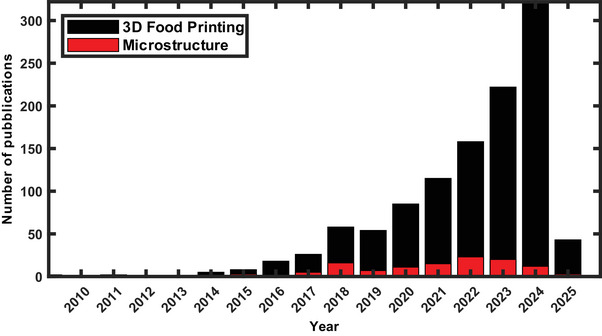
Number of publications about 3D food printing compared with publications reporting the porous structure of 3D‐printed food items over the past two decades, as obtained from scopus.com.

Over the past two decades, the number of publications on 3D food printing has increased markedly, as shown in Figure 1, highlighting the growing academic and industrial interest in this emerging field. Despite this surge in research activity, relatively few studies have focused on the internal porous structure of printed food products (Figure 1). While some investigations have characterized the porosity of 3D‐printed food materials (Koranne et al. 2022; Liu et al. 2020c) or explored its modulation through variations in printing parameters (Derossi et al. 2018, 2022, 2020b, 2023), there remains a significant gap in systematic approaches aimed specifically at controlling porosity. To address this, it is first necessary to establish a clear definition of the types of pores that may be present in such structures. In 3D‐printed materials, internal voids can be broadly categorized into two forms of porosity: inter‐strand and intra‐strand.

**FIGURE 2:**
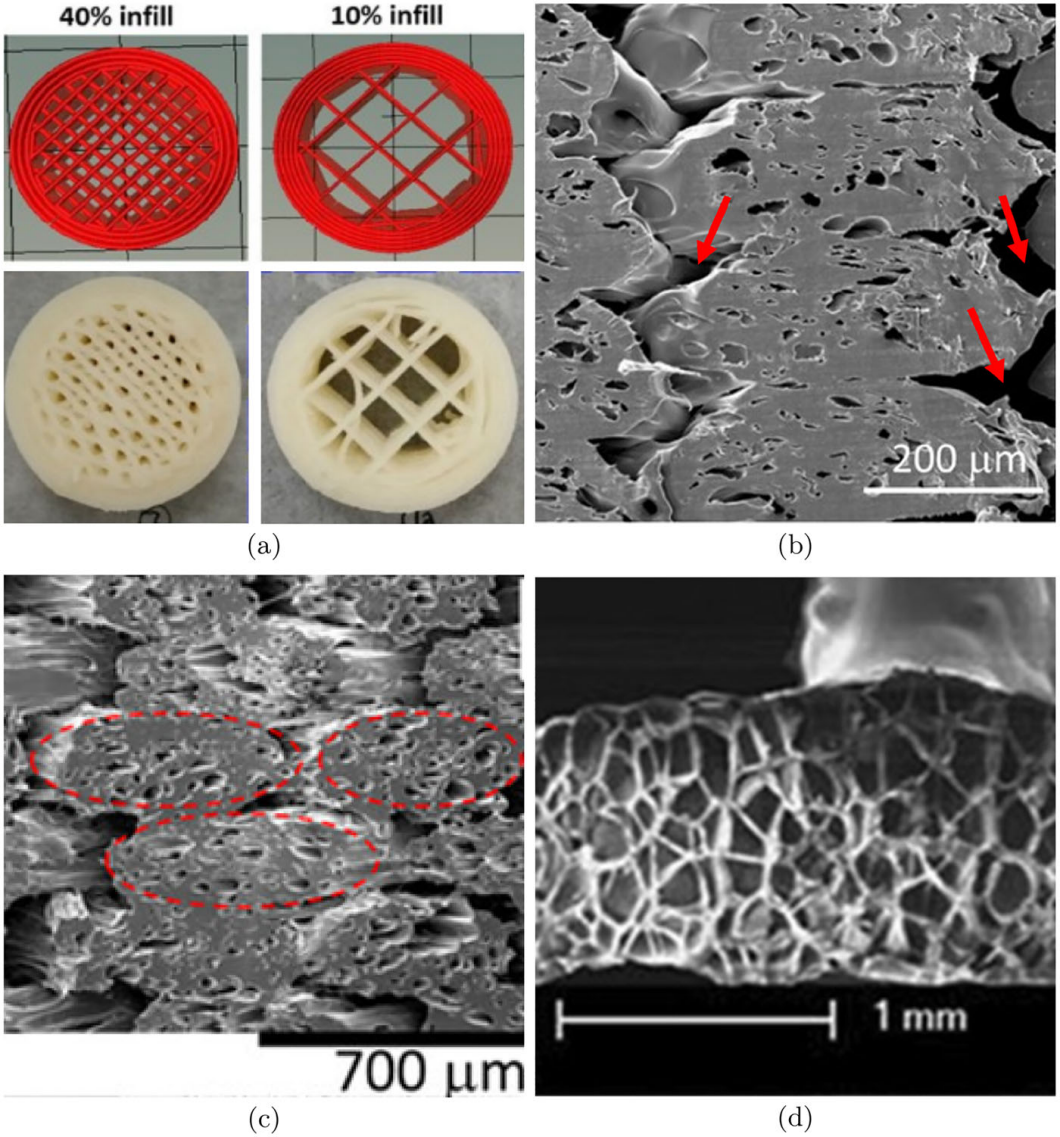
(a) Example of design induced inter‐strand porosity in mashed potatoes 3D printed samples at two different infill percentages. Adapted from Liu et al. (2018b) with permissions from Elsevier. (b,c) SEM images of inter‐strand and intra‐strand pores in 3D printed foamed PLA samples, respectively. Adapted from Tammaro et al. (2022a) with permissions from Wiley. In both cases it is possible to visualize the filament section and where these type of pores are located. (d) SEM detail of intra‐strand pores. Adapted from Marascio et al. (2017) with permissions from Wiley.

Inter‐strand pores form at the interfaces between adjacent strands and typically manifest as macropores, ranging from several hundred micrometers to a few millimeters in size (see Figure 2a,b). These macropores significantly influence the properties of 3D‐printed objects, particularly affecting adhesion and bonding quality (Tammaro et al. 2022a, 2022b). Previous studies have explored the formation of inter‐strand porosity and analyzed how various printing parameters influence their development (Liu et al. 2018b). Key factors governing their formation include layer deposition methods, infill percentage, and the overall design of the 3D model (Bugarin‐Castillo et al. 2023). Macroporosity is essential for maintaining structural integrity and enabling effective load transfer within the printed object.

In contrast, intra‐strand porosity refers to internal pores contained within individual strands (see Figure 2c,d) (Marascio et al. 2017). These micropores are primarily influenced by parameters such as ink composition, printing temperature, cooling rates, and extrusion processes. The presence and characteristics of intra‐strand pores directly affect critical material properties, including mechanical strength, thermal behavior, and structural uniformity of each strand.

Together, inter‐strand and intra‐strand porosity levels interact to shape the overall performance and functionality of 3D‐printed structures. Their combined effect can produce hierarchical pore structures, further enhancing material characteristics (Tammaro et al. 2022a).

The formation of micropores in food‐based materials can be achieved through a wide range of techniques, each influencing the resulting structure and properties in different ways.

**FIGURE 3:**
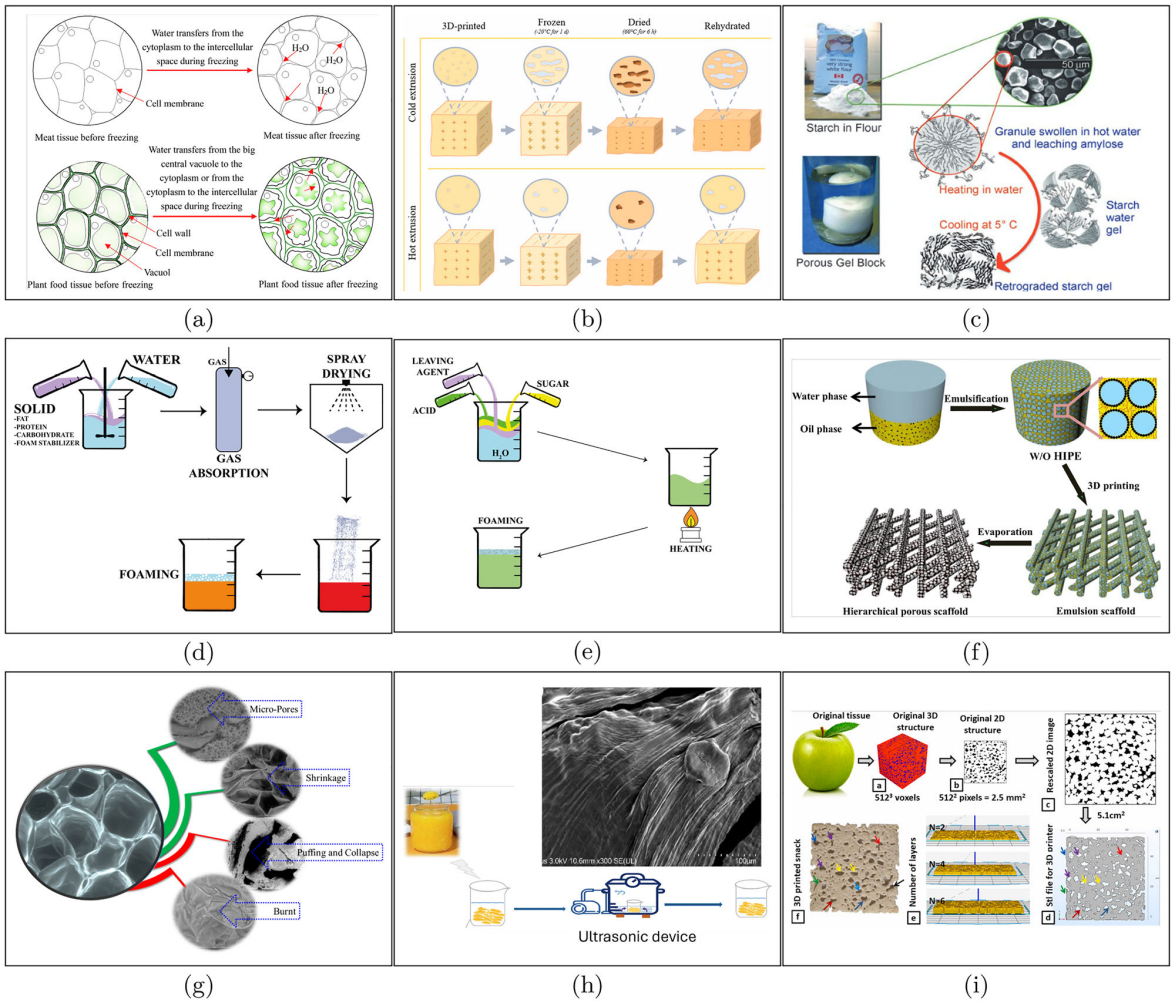
(a) Cell morphology change and water transfer caused by the alteration of osmotic pressure during freezing. Adapted from Li et al. (2018) with permission from Elsevier. (b) Schematic of pores formation during freezing and drying processes in 3D printed meat analogues structure. Adapted from Lombardi et al. (2025b) with permission from Elsevier. (c) Schematics of starch retrogradation mechanism. Adapted from Budarin et al. (2006) with permission from Wiley. (d,e) Illustration of foam creamer production based on inert gas injection and chemical leavening, respectively. Adapted from Aghajanzadeh et al. (2024), with permission from Elsevier. (f) Schematic illustration of the fabrication of bioactive nanoparticle/PCL scaffolds with hierarchical porous structures basing on 3D printing of pickering HIPE templates and volatile phase evaporation. Adapted from (Hu et al. 2019) with permission from Elsevier. (g) Morphological changes that take place during microwave heating of food tissue. Adapted from (Joardder and Karim 2023) with permission from MDPI. (h) Schematic flow diagram of porous structure alteration of orange juice sac induced by ultrasound treatment. Adapted from (Kong et al. 2024) with permission from Springer. (i) Workflow employed for the printing of innovative cereal‐snacks inspired by apple tissue structure. The 3D model includes the pores that have to be present in the final structure. Adapted from (Derossi et al. 2022) with permission from Elsevier.

Freeze‐drying is one of the most commonly used methods for preserving porous structures and protecting them from degradation (Liu et al. 2022a; Li et al. 2018). During the freezing stage, water within the material transitions into ice crystals (Da‐Wen Sun 2005), which can disrupt the porous structure of plant and animal tissues due to physical expansion and osmotic imbalance, as shown in Figure 3a (Li et al. 2018). The rate of freezing plays a crucial role: slow freezing forms large extracellular ice crystals that exert mechanical stress, rupture cell membranes, and lead to tissue damage‐causing increased drip loss and texture softening (Harnkarnsujarit et al. 2015). In contrast, fast freezing results in smaller, more evenly distributed intracellular crystals, which are generally less harmful.

To mitigate structural damage, ice crystals are removed through controlled thawing or vacuum freeze‐drying (Zhao and Takhar 2017; Hu et al. 2021; Duan et al. 2016; Liu et al. 2022a; Ma et al. 2017). Thawing just above 0°C allows gradual melting, reducing disruption. Vacuum freeze‐drying sublimates ice directly from the frozen state, preserving both structure and nutritional quality, making it a preferred method for producing high‐quality dried foods (Duan et al. 2016). The final porous architecture depends on the viscosity of the precursor‐often referred to as the “ink” in structured food systems (Lombardi et al. 2025b). High‐viscosity inks limit crystal growth, producing smaller, more uniform pores, while low‐viscosity systems allow larger crystals and result in larger pores (Figure 3b).

In starch‐based systems, pore formation is driven by retrogradation, a controlled rearrangement of gelatinized starch networks (Ahmadzadeh and Ubeyitogullari 2023a, 2022). Upon heating in water, starch granules release amylose and amylopectin chains (Islam et al. 2024). As the material cools, these chains partially recrystallize into a lamellar structure of crystalline and amorphous regions (Budarin et al. 2006), which serves as a scaffold for pore development (Figure 3c).

Inert gas injection is an effective method to induce porosity in food matrices. This technique involves incorporating gases such as nitrogen or carbon dioxide into a stabilized food matrix, typically facilitated by foaming agents like bovine serum albumin (BSA) (Aghajanzadeh et al. 2024; Vancauwenberghe et al. 2017; Smith et al. 2020; Naik et al. 2023). In foam systems containing volatile compounds dissolved in the liquid phase, stability depends heavily on the delicate interplay among temperature fluctuations, evaporation of volatile substances, and surface‐active species adsorbed at the air‐liquid interface (Lombardi et al. 2023). For instance, BSA functions by stabilizing liquid films between gas bubbles, reducing drainage rates and preventing bubble coalescence, thereby promoting uniform porous structures (Lombardi et al. 2025d, 2025a; Chatzigiannakis et al. 2025; Tammaro et al. 2021). After gas infusion, the mixture undergoes homogenization and spray drying. Upon rehydration, such as when the powder is added to hot liquids like coffee, the entrapped gas is released, resulting in the formation of foams and porous networks (Figure 3d).

Supercritical CO 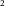 drying offers an alternative to freeze‐drying, particularly for gelatinized starch gels. Operating above CO 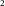 critical point (80 bar, 37°C), this technique removes solvents without collapsing the gel's delicate network (Zou and Budtova 2021). It is especially effective for starches high in amylose, preserving fine, interconnected nanostructures and creating lightweight materials with high surface area and structural integrity (Aghajanzadeh et al. 2024).

In bakery products, chemical leavening is a traditional method for pore formation. Carbon dioxide is released through either thermal decomposition or acid‐base reactions involving sodium bicarbonate (NaHCO 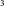) (Aghajanzadeh et al. 2024). Upon heating or exposure to acidic components in the dough, NaHCO 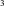 breaks down, producing CO 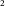 gas, which expands and creates pores within the dough. These are then stabilized during baking, resulting in a light and airy texture (Figure 3e).

Emulsion templating with solvent evaporation is a widely adopted approach in both food and biomaterial science (Li et al. 2024; Shahbazi et al. 2024; Jiang et al. 2022; Liu et al. 2019a; Yu et al. 2022b; Shahbazi et al. 2021b). High internal phase emulsions (HIPEs) or Pickering emulsions (HIPPEs) (Liang et al. 2025), which are typically water‐in‐oil systems stabilized by solid particles (Aghajanzadeh et al. 2024), are commonly employed as structural templates. When the continuous phase solidifies and the dispersed phase evaporates, it leaves behind a microporous network (Figure 3f). Combined with 3D printing, this technique enables the creation of hierarchical scaffolds with both macropores (defined by the printed geometry) and micropores (dictated by the emulsion structure), offering high porosity, tunable morphology, and structural fidelity‐ideal for applications like tissue engineering and functional foods.

External stimuli such as microwave and ultrasound treatments also play a role in pore formation. Microwave‐assisted drying induces vapor pressure within food, leading to the development of nano‐micro‐pores on cell walls (Figure 3g) (Joardder and Karim 2023). Intermittent microwave drying (IMCD) is preferred over continuous drying due to its more uniform heat and moisture distribution, which avoids structural collapse. Ultrasound treatments, meanwhile, influence gel porous structure by promoting protein unfolding and cross‐linking, resulting in a denser and more stable porous network (Figure 3h) (Gu et al. 2024; Li et al. 2025; Kong et al. 2024). However, excessive ultrasound can lead to over‐aggregation and reduced material processability.

A critical distinction exists between spontaneous pore formation, which arises from the intrinsic behavior of food inks during post‐processing, and digitally controlled pore design, which is achieved through CAD modeling and slicing software. While the former governs intra‐strand and inter‐strand microporosity, the latter allows for inter‐strand macropore programming via toolpath design and infill parameters. Combining both strategies offers the potential for hierarchical porous structures with tailored texture and functionality.

For instance, pore formation can be precisely programmed using 3D design tools. Derossi et al. (2022) demonstrated that microtomographic images of apple tissue can be converted into CAD models, which are then used to guide 3D printing of cereal‐based snacks (Figure 3i). By controlling parameters such as internal geometry and layer distribution, this method enables accurate replication of porous architectures and tailored textural properties.

Achieving high‐fidelity porous architectures through digital design is challenging when using complex food materials such as emulsions, protein‐polysaccharide blends, or fibrous pastes. These systems often exhibit rheological behaviors—such as shear thinning, viscoelasticity, or phase separation—that may compromise print resolution and pore fidelity. However, by optimizing ink composition and incorporating post‐processing strategies like freeze‐drying or supercritical CO 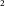 drying, it is increasingly possible to realize digitally modeled pore networks even with structurally delicate formulations.

Among all these methods, freeze‐drying and emulsion templating with solvent evaporation are the most widely adopted techniques in the literature for generating internal porous structures in 3D‐printed food materials. Therefore, this review will primarily focus on these two approaches, analyzing the impact of ink composition and printing parameters. Nonetheless, the other methods remain valuable and offer complementary strategies for designing and optimizing porous food structures. Notably, these techniques also open avenues for the deliberate design of hierarchical porous architectures, which combine macro‐scale voids formed by the printed geometry (inter‐strand pores) with micro‐ or nano‐scale porosity generated within individual filaments (intra‐strand pores) during post‐processing. Such hierarchical designs, well known in materials science, can enhance mechanical stability, improve moisture management, and offer more nuanced control of texture and mouthfeel‐all while using less raw material. By strategically tuning both the printing path and the formulation or processing conditions (e.g., emulsion stability or freezing kinetics), it is possible to engineer multiscale porosity that mimics the complexity of natural food structures. This integrated design approach not only improves functional and sensory properties but also supports the development of more sustainable, low‐density food products without compromising consumer acceptance.

## 3 Formulation Effect

The specific ingredients and their proportions in 3D printing inks play a crucial role in determining the porous structure of the resulting food materials, and this is fundamentally linked to how the pores are formed. For instance, a variation in the protein content of the ink may lead to entirely different effects depending on whether the pores are formed through freeze‐thawing or as a result of emulsion formation. This section aims to examine the influence of ingredients on the porous structure, considering the specific pore formation method used.

### 3.1 Polysaccharides

The selection of ingredients and the specific formulation of printing inks are critical factors influencing the final porous structure of 3D‐printed samples. In particular, polysaccharide‐based inks are often associated with freeze‐thawing processes, which serve as a primary mechanism for pore generation. Table 1 presents selected studies on porous structures in polysaccharide‐based 3D‐printed food materials.

**TABLE 1:** Porous structures in polysaccharides based 3D printed food materials.

Formulation	Process parameters	Main findings	Porous structure SEM	Ref.
Potato starch	Printing velocity: 30 mm/s; Temp.: 60°C, 65°C; Nozzle: 0.8 mm	As the concentration of PS increased, the cell size of the porous structure decreased, and the cell walls became thicker, resulting in a higher cell density	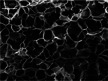	(Liu et al. 2020c)
High amylose corn starch, ethyl cellulose, Lutein	Printing velocity: 4 mm/s; Temp.: 55°C, 65°C, 75°C; Nozzle: 0.7 mm	Corn starch typically requires high‐temperature extrusion to form a well‐defined porous network	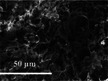	(Ahmadzadeh and Ubeyitogullari 2023a)
Yam starch, guar gum (GG), xanthan gum (XG), carrageenan gum (CG), chitosan (CS), gum Arabic (GA), β‐carotene microcapsules	Printing velocity: 25 mm/s; Temp.: 25°C; Nozzle: 0.6 mm	Hydrocolloids significantly influence the porous structure and printability of yam starch hydrogels, with guar gum and chitosan enhancing uniformity and stability, while carrageenan leads to brittle, less printable structures	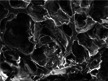	(Feng et al. 2022)
corn starch (CS), rice starch (RS)	Printing velocity: 30 mm/s; Temp.: 65°C–85°C; Nozzle: 0.8 mm	Increasing starch concentration and printing temperature generally reduces pore size and enhances network compactness, improving mechanical strength‐though excessive heat can destabilize the gel structure	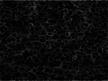	(Zeng et al. 2021)
Soy protein isolate (SPI), acetylated starch, octenyl succinic anhydride starch, ethyl (hydroxyethyl) cellulose, dodecenyl succinylated inulin	Printing velocity: Not provided; Temp.: 25°C; Nozzle: Not provided	Biosurfactants like EHEC, OSA starch, acetylated starch, and dodecenyl succinylated inulin enhance interfacial stability by reducing interfacial tension and increasing disjoining pressure, thereby preventing droplet coalescence	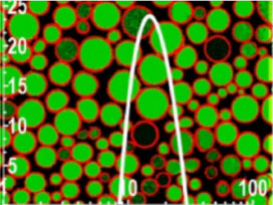	(Shahbazi et al. 2021b)

**FIGURE 4:**
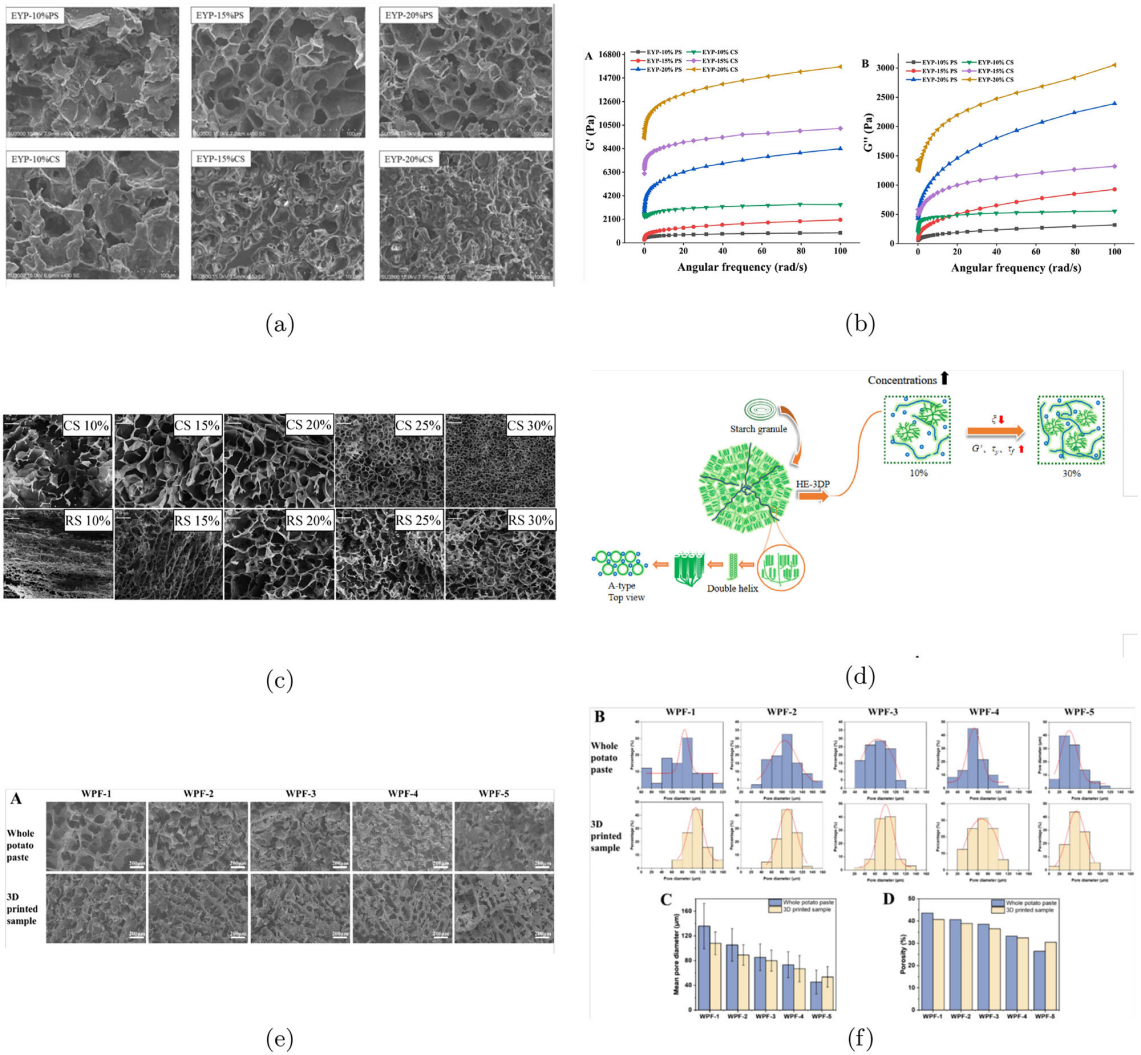
(a,b) SEM images of different egg yolk powder‐starch gels (450× magnification) and small amplitude oscillatory shear (SAOS) behavior and apparent viscosity of gels: Elastic/storage modulus ($G^{\prime }$), viscous/loss modulus ($G^{\prime \prime }$). Adapted from Zhong et al. (2024a) with permissions from Elsevier. (c,d) SEM images of extrusion‐printed corn starch (CS) and rice starch (RS) at different starch content, and schematic representation for the changes of multi‐scale structure and rheological properties of CS and RS at different starch concentrations. Adapted from Zeng et al. (2021) with permissions from Elsevier. (e,f) SEM images, surface pore diameter distribution histograms, and corresponding porosity values of whole potato flour paste and 3D‐printed samples prepared with varying ink moisture contents, which reflect differences in flour chain length. Adapted from Wang et al. (2024) with permissions from Elsevier.

Potato starch, whether in its pure form or as part of whole potato flour, has been widely studied for its role in forming micropores ranging from 1 to 100 μ m. Liu et al. demonstrated that increasing potato starch concentration leads to smaller pore sizes and thicker cell walls, resulting in a denser internal network with enhanced mechanical strength (Liu et al. 2020c; Dankar et al. 2018). Similar behavior has been observed in formulations containing both potato starch and egg yolk powder, where higher starch content correlates with decreased porosity and a more compact porous structure, as shown in Figure 4a (Zhong et al. 2024a, 2024b). Additionally, an increase in water content in potato flour has been shown to extend the average carbohydrate chain length, producing a highly porous structure (Figure 4e) (Wang et al. 2024). Even slight increases in chain length were found to shift the pore size distribution toward smaller diameters in both untreated and 3D‐printed samples (Figure 4f).

Comparable porous structures have also been achieved using corn starch‐based inks. However, in contrast to potato starch, corn starch generally requires extrusion at elevated temperatures to produce a well‐defined porous network (Ahmadzadeh and Ubeyitogullari 2023a, 2022; Zeng et al. 2021). Similar to potato starch, increasing corn starch concentration results in reduced pore size and greater structural compactness, as shown in Figure 4c (Zeng et al. 2021). The amylose content of corn starch is also a key determinant of porosity: high‐amylose starch (72%) yields aerogels with finer, nanoscale porous networks compared to lower amylose variants (55% and 25%) (Ahmadzadeh and Ubeyitogullari 2023b). These high‐amylose materials exhibit superior porosity and specific surface area, enhancing their effectiveness as delivery matrices for bioactive compounds. Furthermore, Johansson et al. observed that samples with higher carbohydrate content‐particularly those rich in starch and fiber‐tended to exhibit irregular internal architectures, often characterized by perforation‐like features. This suggests a trade‐off between carbohydrate concentration and structural regularity (Johansson et al. 2022).

Carbohydrates in the form of gelatin have also been employed to modify porosity independently (Yun et al. 2022; Bulut and Candoğan 2022; Liu et al. 2020b; Chen et al. 2022). At low gelatin concentrations, samples displayed rough surfaces and fragmented, non‐uniform cross‐sections. With increasing gelatin content, the structures became more homogeneous, exhibiting circular intra‐strand pores and thicker walls. These thicker walls contributed to a denser and more mechanically stable network as gelatin concentration increased.

Among polysaccharides, gums are essential functional ingredients in 3D food printing, used to fine‐tune the porosity, and texture of printed products. Commonly used gums include guar gum (GG), xanthan gum (XG), gum Arabic (GA), kappa‐carrageenan (KG), and locust bean gum (LBG). These are often added to polysaccharide‐protein formulations to enhance mechanical stability and surface quality (Azam et al. 2018; Liu et al. 2018b; Pant et al. 2021; Oliveira et al. 2021; Varghese et al. 2020; Dick et al. 2020).

Each gum affects the printed structure differently. For example, in β‐carotene‐loaded yam starch inks, GG and chitosan (CS) promoted uniform porosity and enhanced gel elasticity (Feng et al. 2022). Carrageenan gum (CG) created large, fragile pores, making it less suitable for precise designs. In mashed potato formulations, KG produced thick‐walled porous networks that boosted strength but compromised print smoothness, while XG enabled finer, honeycomb‐like pores and smoother textures. A KG‐XG blend offered a balance between rigidity and print resolution (Liu et al. 2018c). Further, gums impact emulsion and starch‐based gel printing performance. SPI‐GG gels had large, irregular pores, while SPI‐XG formed tighter, denser networks (Yu et al. 2022b). High concentrations of gums generally reduced porosity, improving hardness and compactness. However, excessive LBG caused brittleness and disrupted flow, leading to cracks and printing defects (Yu et al. 2022a). Overall, the type and concentration of gums critically determine the pore structure, mechanical strength, and printability of 3D‐printed food systems, enabling the design of customized textures and structures for specific applications.

Although an increase in carbohydrate concentration in 3D printing inks consistently leads to greater porosity and a noticeable reduction in average pore size, the underlying mechanisms remain a topic of debate across the literature. Zeng et al. (2021) suggest that higher starch concentrations promote the formation of denser gel networks by providing more free molecular chains. These chains can interact through cross‐linking and hydrogen bonding (Figure 4d), resulting in a tighter network with smaller pores and thicker walls, thereby enhancing the gel's mechanical strength and structural integrity. A key factor in this process is the formation of crystalline structures. Rice starch (RS), for instance, develops V‐type crystallinity at higher concentrations and optimal printing temperatures, contributing to a degree of structural inhomogeneity that further strengthens the gel. In contrast, corn starch (CS) generally forms more homogeneous, amorphous networks without new crystalline features, as illustrated in Figure 4c (Zeng et al. 2021).

Supporting this, Wang et al. (2024) propose that the emergence of porous networks is primarily driven by the entanglement of starch molecular chains, which organize into interconnected pore structures. These structural transformations directly influence the ink's rheological properties‐specifically, increasing the storage modulus ($G^{\prime }$), loss modulus ($G^{\prime \prime }$), and overall viscosity (Figure 4b) (Zhong et al. 2024b; Zeng et al. 2021). Such rheological changes significantly impact ice crystal formation during post‐processing. High‐viscosity inks tend to resist ice crystal growth due to their robust structure, leading to smaller, more evenly distributed pores. Conversely, lower‐viscosity inks allow for more extensive ice crystal development and coalescence, producing larger, less uniform pores.

Beyond freeze‐thaw effects, starch retrogradation during cooling also plays a role, especially in high‐temperature treatments typical for corn starch systems (Ahmadzadeh and Ubeyitogullari 2023a, 2022; Zeng et al. 2021). Retrogradation involves the reassociation and partial recrystallization of amylose and amylopectin chains that are released during gelatinization in water (Islam et al. 2024). Upon cooling, these chains reorganize into lamellar structures comprising both crystalline and amorphous regions (Budarin et al. 2006), which form a scaffold that supports pore formation (Figure 3c).

Porous structures formed from emulsions or Pickering emulsions commonly utilize polysaccharides‐such as waxy maize starch‐as the non‐volatile phase. During processing, the evaporation of volatile components like solvents or oils leaves behind a porous matrix (Shahbazi et al. 2024). While these systems are widely used, the influence of polysaccharide concentration on emulsion stability remains underexplored in current literature.

**FIGURE 5:**
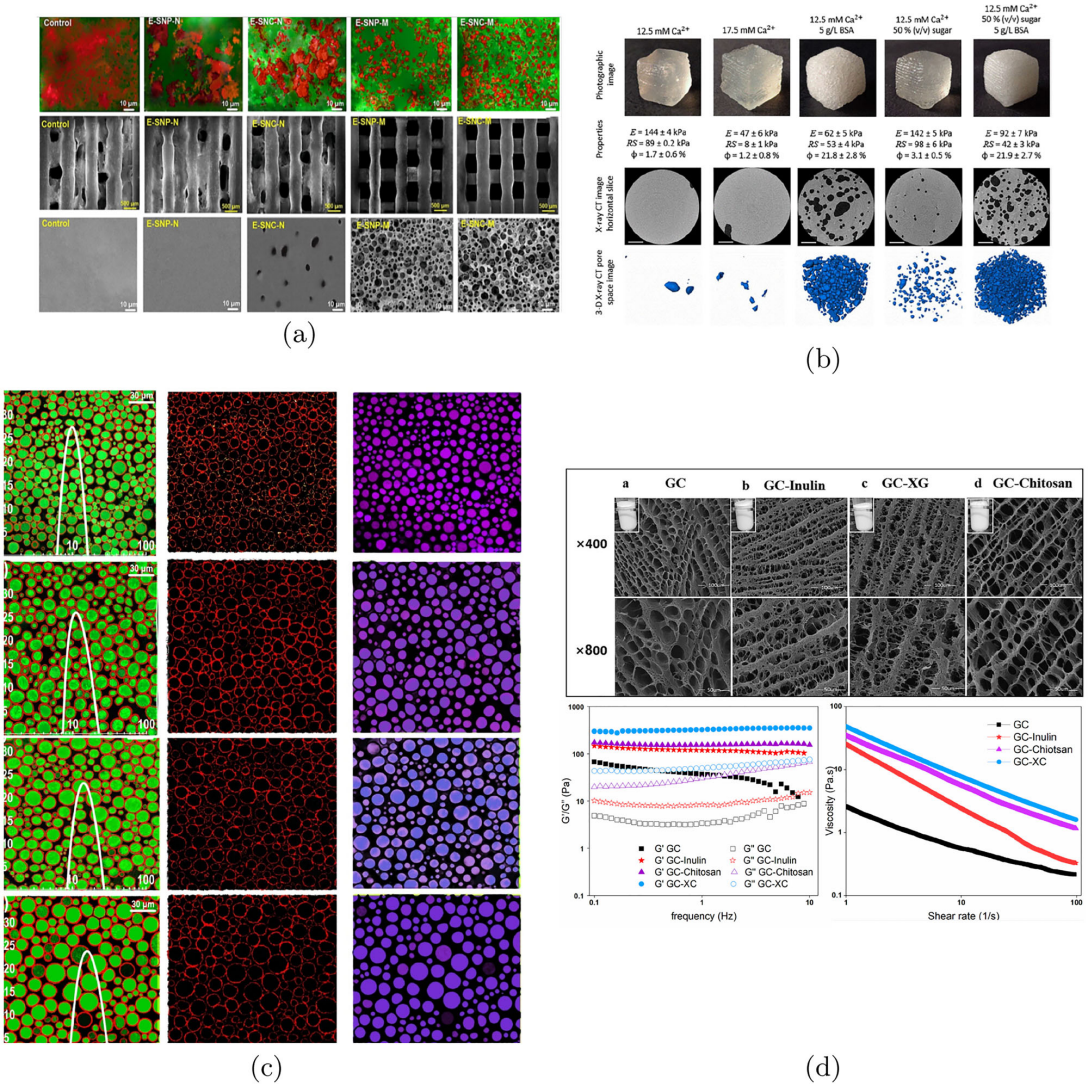
(a) Row i: CLSM images of different non‐freeze‐dried 3D structures. Row ii: Representative images of a one‐layer of freeze‐dried 3D printed‐grid. Row iii: FE‐SEM images of freeze‐dried 3D printed selfsupporting scaffolds. Adapted from Shahbazi et al. (2024) with permissions from Elsevier. (b) Representative samples at macro and micro scales, Young's modulus (E), rupture stress (RS) and porosity (ϕ) of 3‐D printed gels having a pectin concentration of 15 g/L. Scale bar of micro CT images = 1 mm. Adapted from Vancauwenberghe et al. (2017) with permissions from Elsevier. (c) CLSM images of SPI‐based inks stabilized by, Row i: EHEC, Row ii: DS inulin, Row iii: OSA starch, and Row iv: acetylated starch. Oil phase (right column) (for better clarification the green color was changed to purple–blue), protein/biosurfactants stained red (middle column), and overlapping images (left column). Adapted from Shahbazi et al. (2021b) with permissions from Elsevier. (d) Microscopic morphology and frequency sweeps, and apparent viscosity of freeze‐dried protein stabilized Pickering emulsion with different polysaccharides. Adapted from Li et al. (2024) with permissions from Elsevier.

In simple emulsions, polysaccharide‐based surfactants are critical for stabilizing oil‐in‐water systems designed for 3D food printing (Shahbazi et al. 2021b). Biosurfactants such as ethyl(hydroxyethyl) cellulose (EHEC), OSA starch, acetylated starch, and dodecenyl succinylated inulin improve interfacial stability by lowering the interfacial tension and increasing disjoining pressure between two droplets interfaces preventing their coalescence (Figure 5c). This mechanism prevents droplet coalescence and facilitates the development of a cohesive, viscoelastic network that enhances the mechanical strength, rheological behavior, and printability of the emulsion‐key factors in preserving shape during extrusion‐based 3D printing.

In Pickering emulsions, stabilization is achieved through solid particles such as cellulose nanocrystals or nanoparticles, which adsorb at the oil–water (O/W) or water–oil (W/O) interface. Increasing the concentration of these stabilizing particles has been shown to improve emulsion stability (Shahbazi et al. 2024, 2022), as shown in Figure 5a. This typically results in a greater number of dispersed droplets, leading to a finer porous structure with higher porosity and reduced average pore size in the final material.

### 3.2 Proteins

Proteins are extensively employed in 3D printing formulations, primarily to develop meat analogues and stabilize complex multiphase inks, such as foams and emulsions.

In 3D‐printed meat analogues, proteins fulfill a dual role: enhancing nutritional value and providing structural support to ensure better printability and final product stability. High concentrations of proteins, such as soy protein or pea albumin, promote tighter molecular packing. This results in reduced porosity, improved mechanical strength, and enhanced shape fidelity (Shi et al. 2023; Zhou et al. 2023). Additionally, elevated protein levels enhance gluten polymerization, leading to a more robust gluten network, as evidenced by increased viscoelastic modulus values ($G^{\prime }$ and $G^{\prime \prime }$) (Zhang et al. 2018).

Water content significantly influences the porous structure of protein‐rich pastes. Higher moisture levels can disrupt the gel network by breaking disulfide bonds and glutenin interactions, ultimately increasing porosity (Hussain et al. 2022).

Generally, increased protein content raises both $G^{\prime }$ and $G^{\prime \prime }$, making the inks more resistant to deformation. This resistance helps prevent the growth of large ice crystals during freezing, thus reducing final pore sizes. However, Liu et al. (2018a) observed an opposite trend when combining milk protein concentrate (MPC) and whey protein isolate (WPI). Their study found that the most porous structures corresponded to the lowest viscoelastic modulus values. An optimal MPC:WPI ratio of 5:2 resulted in a cohesive, elastic, and homogeneous matrix, enhancing extrusion smoothness and layer adhesion due to improved hydration, water distribution, and protein particle interactions.

Proteins also influence porosity through interactions with other components. For example, combining proteins and saccharides during cooking or heating can trigger the Maillard reaction (Wen et al. 2022). This reaction forms stable conjugates between saccharides and proteins, promoting cross‐linking and resulting in a denser protein network. Consequently, mechanical properties and texture in meat analogues improve. Additionally, protein denaturation plays a crucial role by increasing ink viscosity, thus hindering ice crystal growth and pore formation during freezing (Lombardi et al. 2025d; Zhong et al. 2024a).

As previously discussed, proteins are essential stabilizers of emulsions and foams. In foam systems, proteins like bovine serum albumin (BSA) act as surface‐active agents, migrating to the air–liquid interface of air bubbles to reduce surface tension and prevent bubble coalescence, stabilizing the foam structure (Vancauwenberghe et al. 2017). Studies have particularly explored BSA's film‐forming capabilities, critical for stabilizing thin liquid films between bubbles (Lombardi et al. 2025d, 2025a; Chatzigiannakis et al. 2025).

In Pickering emulsions, proteins function as particulate stabilizers, adsorbing at oil–water interfaces to create a solid‐like barrier, thus preventing droplet coalescence (Dhara et al. 2024; Liang et al. 2025). An exemplary application includes gelatin‐catechin nanoparticles (GCNPs) used to stabilize oil‐in‐water emulsions for 3D printing (Li et al. 2024). These emulsions exhibit improved structural integrity, water retention, and rheological behavior critical for printability.

Combining protein‐based emulsions with polysaccharides further enhances their stability and rheological performance. For instance, inulin, a neutral polysaccharide, engages in weak hydrogen bonding with proteins, resulting in moderately stabilized emulsions (Li et al. 2024). Xanthan gum, an anionic polysaccharide, creates strong electrostatic repulsion with negatively charged GCNPs, enhancing gel strength. Conversely, positively charged chitosan strongly interacts electrostatically with proteins, forming a thick, rigid gel network. This interaction significantly improves emulsion stability and structural precision of the 3D‐printed products.

Incorporating polysaccharides modifies emulsion porosity and increases storage ($G^{\prime }$) and loss moduli ($G^{\prime \prime }$), reinforcing the solid‐like and shear‐thinning properties critical for successful extrusion‐based 3D printing. It has also been observed that increasing the size of dispersed volatile droplets encourages the formation of open‐cell structures. Larger droplets evaporate more readily during drying, creating interconnected pores, a phenomenon similarly observed in high‐moisture systems (Li et al. 2024).

The formulation's impact on porous structures in 3D‐printed food remains complex due to various ingredient combinations. Nonetheless, a general trend emerges: higher concentrations of polysaccharides or proteins typically result in reduced porosity, yielding denser, mechanically stable structures. Despite this understanding, detailed characterization of pore size, density, and bulk properties remains limited but is crucial for optimizing the design and functionality of 3D‐printed foods. Table 2 summarizes several studies examining the influence of proteins on porous structure, with further details provided.

**TABLE 2:** Porous structures in protein based 3D printed food materials.

Formulation	Process Parameters	Main Findings	Porous Structure SEM	Ref.
Aronia melanocarpa fruit pulp, methylcellulose (MC), pea albumin (PA), hyaluronic acid (HA)	Printing velocity: 30 mm/s; Temp.: Not provided; Nozzle: 1.20 mm	Pea albumin (PA) improved gel compactness and, with hyaluronic acid (HA), formed a stable porous structure for printing, but excess PA reduced printability and too much HA caused uneven porosity and deformation.	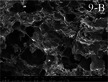	(Zhou et al. 2023)
Milk protein concentrate (MPC), Whey protein isolate (WPI), glycerol, xanthan gum, water	Printing velocity: 35 mm/s; Temp.: 25°C; Nozzle: 0.84 mm	The network's porosity decreased and internal structure became more uniform as wheat protein isolate (WPI) content increased, with uniformity improving in proportion to WPI levels.	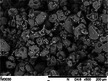	(Liu et al. 2018a)
Mung bean protein isolate (MBPI), methylcellulose, beet red (BR), D‐xylose	Printing velocity: 30 mm/s; Temp.: 25°C; Nozzle: 1.10 mm	Increasing xylose concentration enhances Maillard reactions, leading to more cross‐linking and a denser protein network that improves the texture and strength of meat analogs.	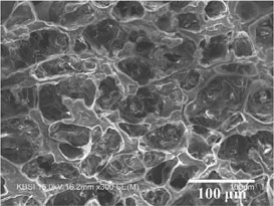	(Wen et al. 2022)
Soy protein isolate, red cabbage powder, glycerol, sodium alginate	Printing velocity: 15 mm/s; Temp.: 21°C; Nozzle: 0.84 mm	Soy protein isolate (SPI) concentration influences pore structure in 3D‐printed food, with 25% SPI offering optimal printability and porosity, while higher levels increase viscosity, reducing accuracy and pore formation.	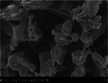	(Carranza et al. 2023)
Protein‐rich, starch‐rich, fiber‐rich faba bean fractions, water	Printing velocity: Not provided; Temp.: 25°C; Nozzle: 0.58 mm	Protein‐rich and protein‐starch formulations created smoother, denser structures with fewer pores, whereas starch‐ and fiber‐rich samples had more open porosity, and the absence of fiber led to structural collapse, emphasizing fiber's role in maintaining porous integrity.	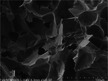	(Johansson et al. 2022)

### 3.3 Lipids

Lipids, typically present as the dispersed oil phase in emulsions, play a crucial but often underappreciated role in shaping the porous architecture of 3D‐printed materials. In emulsion‐templated inks, oil droplets act as sacrificial templates: once the continuous phase solidifies and the lipid phase is removed‐through evaporation, solvent extraction, or freeze‐drying‐a microporous network remains (Liang et al. 2025). The size and distribution of these lipid droplets govern the final pore structure, with finer dispersions yielding higher porosity and smaller, more uniform pores (Shahbazi et al. 2024, 2022).

Emulsion stability depends on interfacial design: polysaccharide‐based surfactants, such as OSA starch or dodecenyl succinylated inulin, stabilize oil‐in‐water systems by reducing interfacial tension and enhancing disjoining pressure, thereby preventing droplet coalescence during extrusion (Shahbazi et al. 2021b). In Pickering emulsions, stabilization is instead achieved by solid particles or protein‐polyphenol complexes, such as GCNPs, which form rigid shells around droplets and improve structural integrity (Li et al. 2024). The chemical composition of the lipid phase also matters: oils with low melting points may drain before solidification, producing open‐cell networks, while fats with high melting points crystallize early, reinforcing the matrix and favoring closed‐cell structures (Liang et al. 2025).

Proteins at the oil–water interface can further interact with oxidized lipids or polysaccharides, enhancing cross‐linking and structural cohesion (Dhara et al. 2024), while antioxidant‐rich stabilizers like GCNPs help mitigate lipid oxidation and improve interface durability (Li et al. 2024). From a nutritional and environmental perspective, emulsion‐templated printing enables the temporary use of lipids to create texture without contributing fat to the final product, and it facilitates the upcycling of low‐value oils, supporting sustainable formulation strategies (Aghajanzadeh et al. 2024).

## 4 3D Food Printing Process Parameters

Several studies have concentrated on optimizing key parameters in 3D food printing, including printing temperature, velocity, flow rate, and nozzle diameter. Adjusting these parameters is crucial to producing food products that meet the desired textural and aesthetic standards, maintain nutrient content, and enable scalability in food production.

### 4.1 Printing Temperature

Printing temperature plays a particularly significant role in 3D food printing as it directly affects the flow properties, texture, and structural integrity of the final product. Different materials such as chocolate, dough, cheese, and vegetable purees require specific temperature ranges for optimal results. Precise temperature control is especially critical for ingredients sensitive to degradation at higher temperatures.

Despite its importance, most studies in the literature report performing 3D food printing at room temperature, typically between 20°C and 25°C, without thoroughly investigating the effects of temperature (Li et al. 2024; Xu et al. 2023; Wei et al. 2024; Wang et al. 2024; Yun et al. 2022; Bulut and Candoğan 2022; Johansson et al. 2022; Liu et al. 2019a; Dick et al. 2020; Wang et al. 2018; Feng et al. 2022; Carranza et al. 2023; Liu et al. 2020b; Wen et al. 2022; Yu et al. 2022b; Zhong et al. 2024a; Liu et al. 2018b; Shi et al. 2023; Montoya et al. 2021; Liu et al. 2018a; Zhong et al. 2024b; Pant et al. 2021; Lee et al. 2019; Vancauwenberghe et al. 2017; Shahbazi et al. 2021b; Derossi et al. 2020a; Vancauwenberghe et al. 2018; Derossi et al. 2020b, 2018; Guénard‐Lampron et al. 2021; Oliveira et al. 2021). In these cases, the only significant temperature changes experienced by the printed samples occur during freezing, with reported temperatures ranging from −18°C to −100°C (Johansson et al. 2022; Lee et al. 2019; Xu et al. 2023; Dick et al. 2020; Ahmadzadeh and Ubeyitogullari 2023b), or during drying, which is typically performed at 20°C to 50°C (Wang et al. 2024; Lee et al. 2019; Ahmadzadeh and Ubeyitogullari 2023b).

Freeze‐drying, also known as lyophilization, is a widely used post‐processing technique for 3D‐printed food samples, aimed at enhancing their preservation and functionality. This method involves the removal of moisture at low temperatures, ensuring that the intricate structures and designs of the printed food remain intact without deformation or shrinkage (Cotabarren et al. 2023; Ahmadzadeh and Ubeyitogullari 2023b). Additionally, freeze‐drying effectively retains the nutritional content, flavor, and color of food, making it particularly well‐suited for custom‐designed meals tailored to specific dietary or sensory preferences (Zhong et al. 2023). During the freezing stage, ice crystals nucleate and grow within the sample, forming voids whose dimensions are governed by the cooling rate. Slower cooling rates typically result in the formation of larger ice crystals, while faster cooling rates lead to smaller ice crystals (Petzold and Aguilera 2009; Kumar et al. 2020). During the subsequent drying phase, as the ice sublimates, it leaves behind a network of interconnected pores, thereby generating a porous structure in the sample.

**FIGURE 6:**
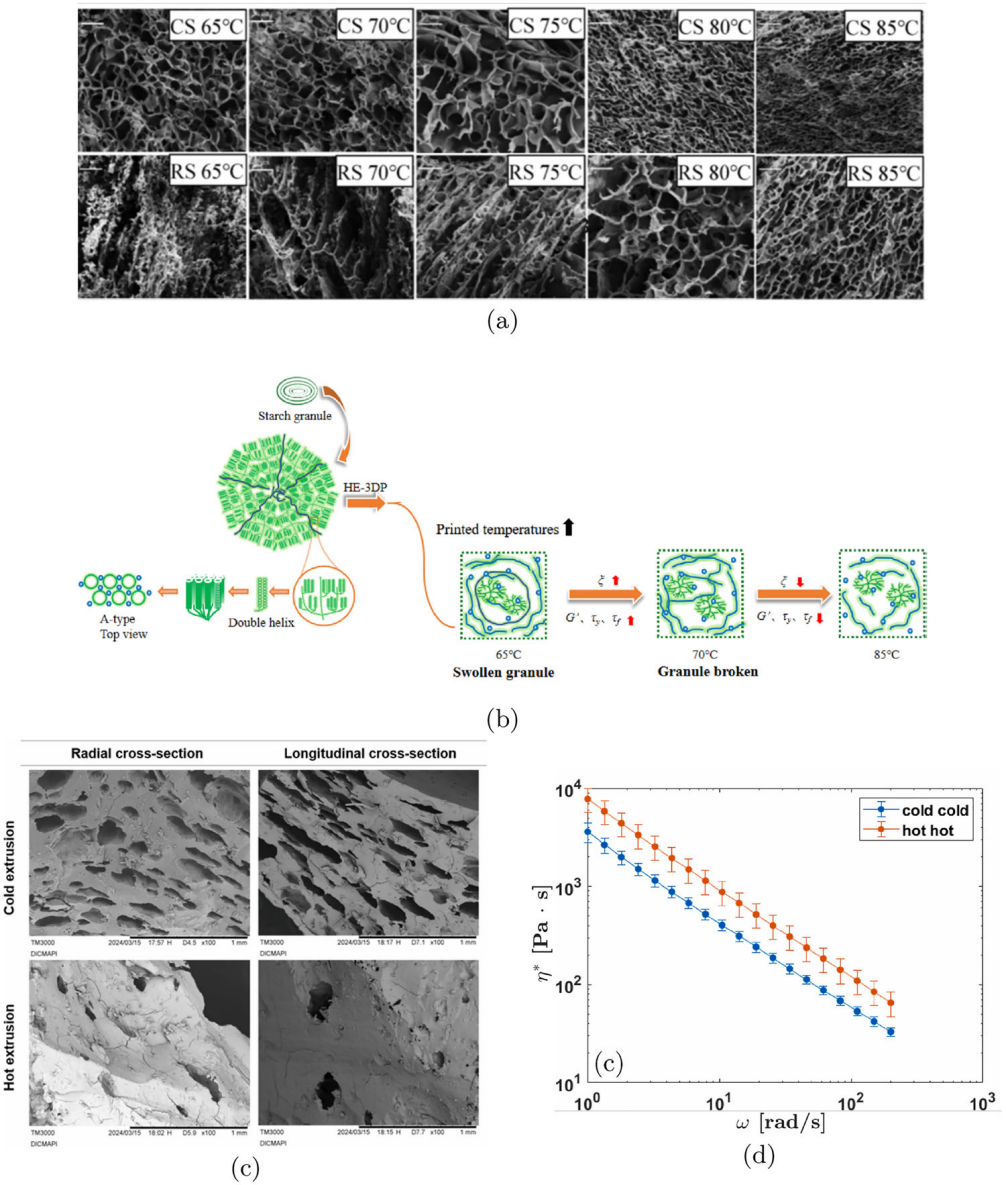
(a,b) SEM images of extrusion‐printed corn starch (CS) and rice starch (RS) at different printing temperatures, and schematic representation for the changes of multi‐scale structure and rheological properties of CS and RS at different printing temperatures. Adapted from Zeng et al. (2021) with permissions from Elsevier. (c,d) SEM micrographs of radial cross‐section, and longitudinal cross‐section of thawed and dried 3D printed products, for samples prepared in hot (85°C) or cold conditions (room temperature), and complex viscosity as a function of oscillation frequency for samples printed at room temperature after mixing at room temperature, and at 85°C after hot mixing (85°C). Adapted from Lombardi et al. (2025b) with permissions from Elsevier.

Several studies have explored how printing temperature influences the internal porous structure of 3D‐printed samples, particularly in starch‐based materials. At lower temperatures, incomplete gelatinization leads to a loose, poorly structured gel network due to limited unwinding and entanglement of starch chains (Zeng et al. 2021). As the temperature increases, the gel network becomes more compact and uniform. Scanning electron microscopy (SEM) of starch gels (Figure 6a) shows that corn starch (CS) forms a porous network with smaller, thinner pores at higher temperatures, while rice starch (RS) develops a stereoscopic, homogeneous structure in the 75‐85°C range. This densification is attributed to enhanced cross‐linking and molecular entanglement, which significantly improves the mechanical strength of the gel (Figure 6b). The degree of gelatinization‐determined by preheating temperature and duration‐plays a key role in micropore formation (Islam et al. 2024). Gelatinization activates hydroxyl and carboxylic acid groups on amylopectin and cellulose fibers, promoting hydrogen bonding and facilitating pore development. However, at excessively high temperatures (e.g., 85°C), the gel structure can become destabilized, leading to reduced mechanical strength and poorer porous structure quality (Zeng et al. 2021).

A similar temperature‐dependent trend is observed in protein‐based systems. At higher printing temperatures (around 85°C), 3D‐printed samples composed of soy protein or wheat gluten exhibit smoother, more compact structures (Lombardi et al. 2025b). This behavior is primarily attributed to protein denaturation, which occurs at elevated temperatures. Denaturation increases the storage ($G^{\prime }$) and loss moduli ($G^{\prime \prime }$), resulting in higher viscosity. The increased viscosity, in turn, restricts water mobility and inhibits ice crystal formation, contributing to improved structural integrity during and after printing (Fan et al. 2018).

In contrast, at lower temperatures (e.g., 25°C), weaker protein‐particle interactions‐mainly through hydrogen bonding‐lead to rougher, more deformable structures with irregular cross‐sections. This contrast is clearly illustrated in SEM images of non‐denatured (cold‐printed) versus heat‐denatured (hot‐printed) protein gels (Figure 6c) and is further supported by differences in complex viscosity behavior (Figure 6d).

Interestingly, studies have also reported a nonmonotonic rheological response with temperature. As temperature increases below the protein denaturation threshold, a decrease in $G^{\prime }$ and $G^{\prime \prime }$ can occur, leading to softer structures with slightly higher porosity (Chen et al. 2022). This reveals that the effect of temperature on protein‐based inks is not strictly linear but depends on a balance between thermal activation and protein network formation.

A comparable nonmonotonic trend was observed by Liu et al., who examined the impact of printing temperatures from 60°C to 80°C on the porous structure of 3D‐printed potato starch systems (Liu et al. 2020c). They found that increasing the temperature from 60°C to 70°C resulted in smaller and more uniform pores, reflecting a denser and more homogeneous network structure. This improvement was attributed to enhanced starch gelatinization and the corresponding rise in viscosity, which limited ice crystal growth during freezing. However, when the temperature reached 80°C, $G^{\prime }$ decreased after peaking near 65°C, and the pore distribution became more irregular‐likely due to diminished molecular interactions at excessive temperatures.

Comparing studies conducted at different extrusion temperatures can be challenging due to variations in the formulations used. However, when similar formulations are analyzed across significantly different temperatures, consistent trends, such as those outlined above, can be observed. For instance, a comparison of results for corn starch‐based inks printed at 25°C (Zhong et al. 2024a) and those printed at 95°C (Ahmadzadeh and Ubeyitogullari 2022, 2023b) reveals notable differences in pore size. At lower temperatures, the samples exhibit relatively large pores ranging from 20–50 μ m, whereas at higher temperatures, the pore size is considerably reduced, ranging between 1–5 μ m. Several studies on the impact of temperature on porous structure are presented in Table 3 below, along with additional details.

**TABLE 3:** Overview of studies examining how printing temperature influences porosity in 3D‐printed food formulations.

Formulation	Process parameters	Main findings	Porous structure SEM	Ref.
Jackfruit seed powder, finger millet powder, xanthan gum, butter, sugar, baking powder, soy protein isolate	Printing velocity: 50 mm/s; Temp.: 25°C–30°C; Nozzle: 0.5 mm	Higher temperature (30°C) softened dough due to butter melting, reducing pore definition; lower temperature (27°C) maintained firmer structure and clearer porosity.	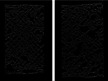	(Varghese et al. 2020)
Soy protein isolate (SPI), sodium alginate, gelatin	Printing velocity: 10 mm/s; Temp.: 25°C–35°C–45°C; Nozzle: 1.55 mm	Nonmonotonic rheological behavior: higher temperature can lead to smoother, denser protein gels; slightly higher porosity at sub‐denaturation temps.	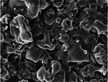	(Chen et al. 2022)
Egg yolk powder, potato starch, guar gum (GG)	Printing velocity: 30 mm/s; Temp.: 25°C; Nozzle: 0.84 mm	Printed at 25°C: relatively large pores (20–50 μ m) in starch‐based ink due to low gelatinization.	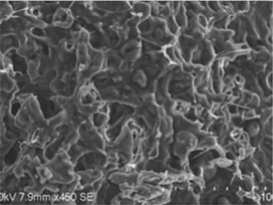	(Zhong et al. 2024a)
Corn starches (25%, 55%, 72% amylose), supercritical carbon dioxide, ethanol	Printing velocity: 6 mm/s; Temp.: 95°C; Nozzle: 0.152 mm	Freeze‐drying creates porous structure; small pores due to high temp (95°C) and high amylose content.	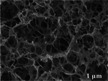	(Ahmadzadeh and Ubeyitogullari 2023b)
corn starch, water	Printing velocity: 6 mm/s; Temp.: 95°C; Nozzle: 0.08–0.33 mm	Printed at 95°C: small pores (1–5 μ m) due to full gelatinization and enhanced molecular interactions.	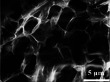	(Ahmadzadeh and Ubeyitogullari 2022)

### 4.2 Printing Velocity

Printing velocity, or the speed of the 3D printer head, plays a critical role in determining layer thickness, product consistency, and overall production efficiency. It is a key parameter in balancing print quality with throughput. To the best of our knowledge, only Derossi et al. have investigated the effect of 3D printing velocity on the internal microstructure of food‐based samples (Derossi et al. 2018). Their findings indicate that printing speed significantly influences filament thickness and the distribution of inter‐strand pores in 3D‐printed food constructs.

**FIGURE 7:**
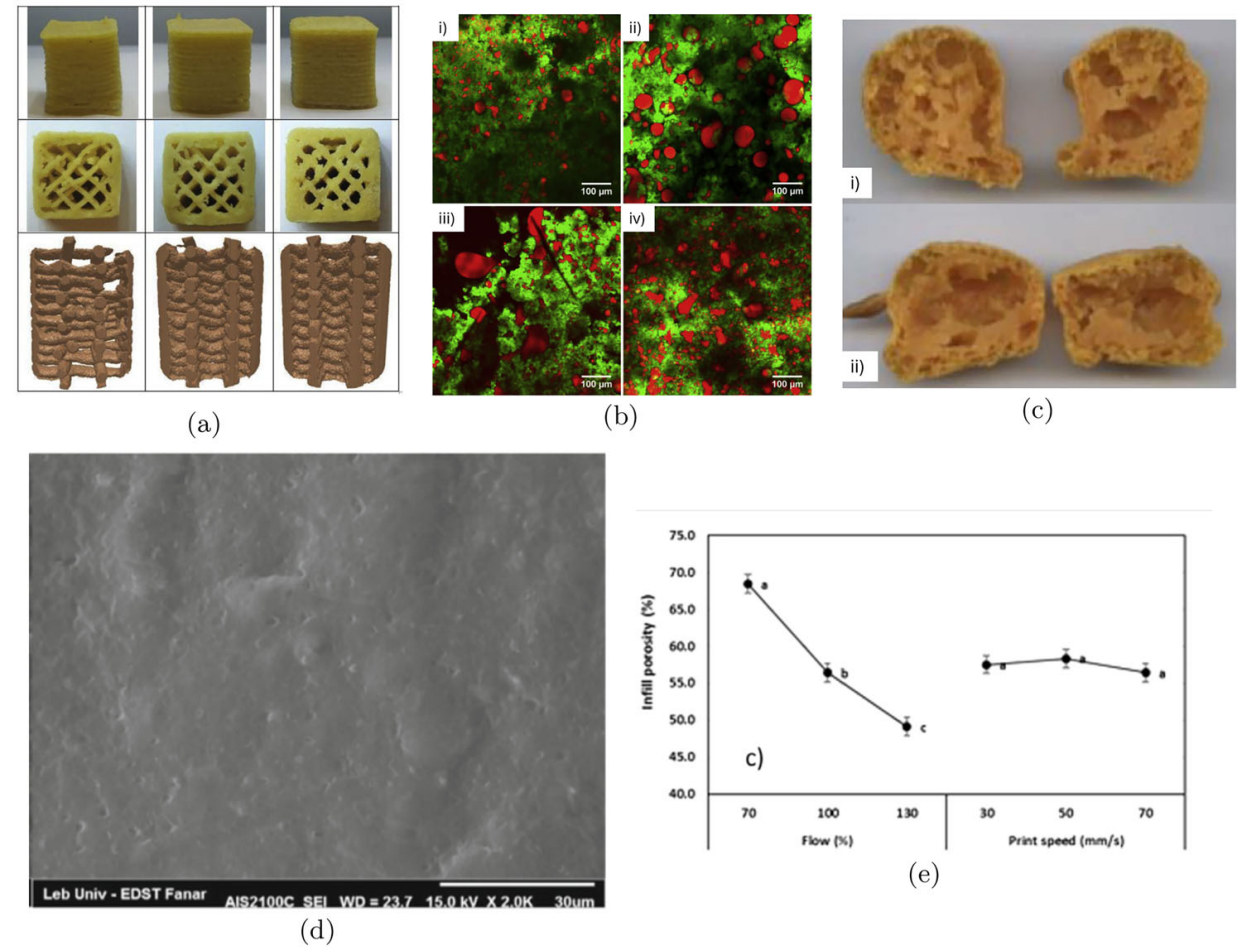
(a,e) Representative pictures of printed snacks and reconstructed 3D X‐ray images (Column i) printing speed 30 mm/s, flow 70%, (Column ii) printing speed 50 mm/s, flow 100%, (Column iii) printing speed 70 mm/s, flow 130%, and main effects of print speed and flow on inter‐strand porosity of printed snacks, adapted from Derossi et al. (2018) with permissions from Elsevier. (b) Confocal laser scanning micrographs of cheese samples stained with Nile Red (lipids) and Fast Green FCF (protein) solutions: (i) untreated cheese, (ii) melted cheese, (iii) low speed processed cheese and (iv) high speed processed cheese. Adapted from Le Tohic et al. (2018) with permissions from Elsevier. (c) Effect of nozzle diameter on carrot based paste internal porous structure: (i) nozzle diameter 3.4 mm and (ii) nozzle diameter 5.1 mm. Adapted from Guénard‐Lampron et al. (2021) with permissions from Elsevier. (d) SEM analysis of potato puree samples processes with a printing speed of 100 mm/s. Adapted from Dankar et al. (2018) with permissions from Springer.

As illustrated in Figure 7a, lower printing speeds (e.g., 30 mm/s) produce thinner and more uniform filaments but result in larger pores, leading to a wider range of pore diameters and increased overall porosity. In contrast, higher speeds (e.g., 50 and 70 mm/s) produce thicker filaments and smaller, more uniformly distributed pores, thereby reducing porosity. However, as shown in Figure 7e, the influence of printing speed on inter‐strand porosity appears to be minimal.

Speed also affects structural uniformity. At lower velocities, material deposition is more consistent, yielding smoother and more regular filament patterns. However, at higher velocities, the increased deposition rate may introduce minor structural irregularities due to insufficient time for material relaxation. Furthermore, the growth rate of the printed sample height increases with speed up to 50 mm/s, but levels off at 70 mm/s, suggesting a saturation point. Overall, higher printing velocities lead to denser, more compact structures with smaller pores and a more refined internal porous architecture.

Comparisons can be made across studies that use different printing velocities but similar formulations. For example, Dankar et al. processed a potato starch‐based ink at a relatively high printing speed of 100 mm/s, which resulted in a poorly developed porous structure with almost no visible pores, except for small ones measuring 1–5 μ m (Figure 7d) (Dankar et al. 2018). In contrast, similar formulations printed at lower velocities (15–30 mm/s) produced a more pronounced porous architecture, with pore sizes ranging from 20–200 μ m (Wang et al. 2024; Zhong et al. 2024a, 2024b).

### 4.3 Extrusion Flow Rate

Flow rate or the extrusion velocity of material from the nozzle, has a direct impact on layer thickness, texture, and the resulting porous structure of 3D‐printed samples.

One common way to express flow rate is as a percentage (Derossi et al. 2018, 2022, 2020b; Cao et al. 2025; Chen et al. 2022), where 100% corresponds to the default extrusion rate recommended by the printer manufacturer. Values above or below 100% indicate increases or reductions in material flow, respectively. Alternatively, flow rate can be quantified in units of volume per time‐such as millimeters per second (mm/s) for filament deposition speed (Dankar et al. 2018), or milliliters per minute (mL/min) for liquid or paste‐like materials common in food and biomedical applications (Shahbazi et al. 2024). Some 3D printing systems display flow rate in terms of steps per second, referring to the stepper motor movement that controls extrusion. Another method for regulating flow involves adjusting the applied extrusion pressure, which directly influences the amount of material extruded through the nozzle (Ahmadzadeh and Ubeyitogullari 2022, 2023a, 2023b; Bulut and Candoğan 2022).

When flow rate is expressed as a percentage, the actual volumetric value is often unspecified, allowing only relative comparisons. Derossi et al. (Derossi et al. 2018) explored flow rates of 70%, 100%, and 130% using a Delta 2040 3D printer. At lower flow levels, printed structures displayed irregularities such as interrupted filament lines and enlarged pores due to under‐extrusion. As flow rate increased, filament deposition became more consistent, resulting in denser, more uniform structures with reduced porosity (Figure 7e). Cross‐sectional X‐ray imaging further confirmed substantial differences in filament thickness and pore distribution across the flow settings (Figure 7a). Higher flow rates produced thicker, more interconnected filaments and a tighter, less porous internal structure, while lower flow rates maintained a more open and loosely connected network.

The effect of extrusion speed also varies by material. For potato starch‐based inks, higher flow rates led to greater porosity and reduced structural uniformity, including larger voids and weaker interlayer connections (Dankar et al. 2018). In contrast, for cheese‐based inks, changes in flow rate had a milder effect on porosity and fat distribution. However, at higher speeds, slightly smaller and more uniformly distributed fat globules were observed, suggesting that increased shear during extrusion can influence the microstructure (Figure 7b) (Le Tohic et al. 2018).

### 4.4 Nozzle Diameter

Nozzle diameter plays a crucial role in shaping the quality of 3D‐printed samples, directly affecting printing precision, surface finish, structural stability, and internal porosity (Wang et al. 2018; Zhang et al. 2018). In the case of wheat‐based inks, larger nozzle diameters tend to produce denser structures with fewer, but larger, pores. In contrast, smaller nozzle diameters result in a more porous internal architecture, as illustrated in Figure 7c (Guénard‐Lampron et al. 2021).

However, using smaller nozzles also increases the required extrusion pressure, which can influence structural integrity. For example, in starch‐mango blends, smaller nozzles cause a greater pressure drop across the nozzle, leading to die swell—a phenomenon where the material expands upon exiting the nozzle. This swelling can reduce printing accuracy and compromise the stability of printed layers. Despite these drawbacks, smaller nozzles can enable the formation of taller structures, thanks to the material more solid‐like behavior under these high‐pressure conditions (Montoya et al. 2021).

In another study, Shi et al. (2023) explored nozzle diameters ranging from 200 to 600 μ m to control the porosity of fish‐analogue samples. They found that smaller nozzles produced denser, more compact filament arrangements with significantly reduced porosity, closely resembling the microstructure of real fish muscle fibers. Although the authors did not elaborate on the underlying mechanism, this effect may be attributed to the extremely fine nozzle diameters used (e.g., 200 μ m), which promote tightly packed deposition and limit pore formation. Table 4 collects the aforementioned studies providing more details.

**TABLE 4:** Overview of studies examining how nozzle diameter influences porosity in 3D‐printed food formulations.

Formulation	Process parameters	Main findings	Porous structure SEM	Ref.
Surimi, NaCl, gelatin, alginate	Printing velocity: 28 mm/s; Temp.: 25°C; Nozzle: 0.80–2.00 mm	Nozzle diameter affects printing quality, precision, surface finish, and porosity. Larger nozzles produce denser structures with fewer but larger pores.	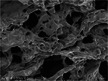	(Wang et al. 2018)
Wheat flour, carrot puree, distilled water	Printing velocity: 10 mm/s; Temp.: 20°C; Nozzle: 2.50–6.00 mm	Smaller nozzle diameters lead to more porous internal architecture in wheat‐based inks.	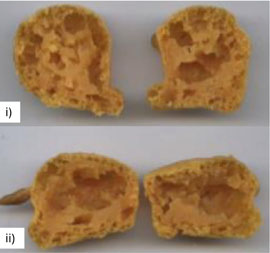	(Guénard‐Lampron et al. 2021)
Starch, mango, arabinoxylans (AX), mineral water	Printing velocity: 3–6–10 mm/s; Temp.: 25°C; Nozzle: 0.40–0.60–1.00 mm	Smaller nozzles increase extrusion pressure, causing die swell and lower stability, but enable taller structures due to solid‐like behavior.	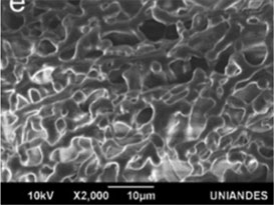	(Montoya et al. 2021)
Soy protein isolate (SPI), xanthan gum (XG), rice starch (RS)	Printing velocity: 20–25 mm/s; Temp.: 25°C; Nozzle: 0.20–0.60 mm	Smaller nozzles reduce porosity and produce compact filaments that resemble real fish muscle microstructure.	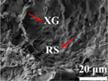	(Shi et al. 2023)

## 5 Influence of Porous Structure on Mechanical Properties

### 5.1 Mechanical Properties

The mechanical properties of 3D‐printed samples are significantly influenced by their internal porous structure. These properties are typically assessed through compression tests, which allow the determination of key parameters such as Young's modulus, fracture stress, and energy absorption (Derossi et al. 2020b; Lombardi et al. 2025b). The relationship between mechanical performance and pore characteristics‐including pore size, quantity, shape, and spatial distribution is complex and non‐linear, as these features interact to determine how stresses are transmitted and dissipated through the material.

**FIGURE 8:**
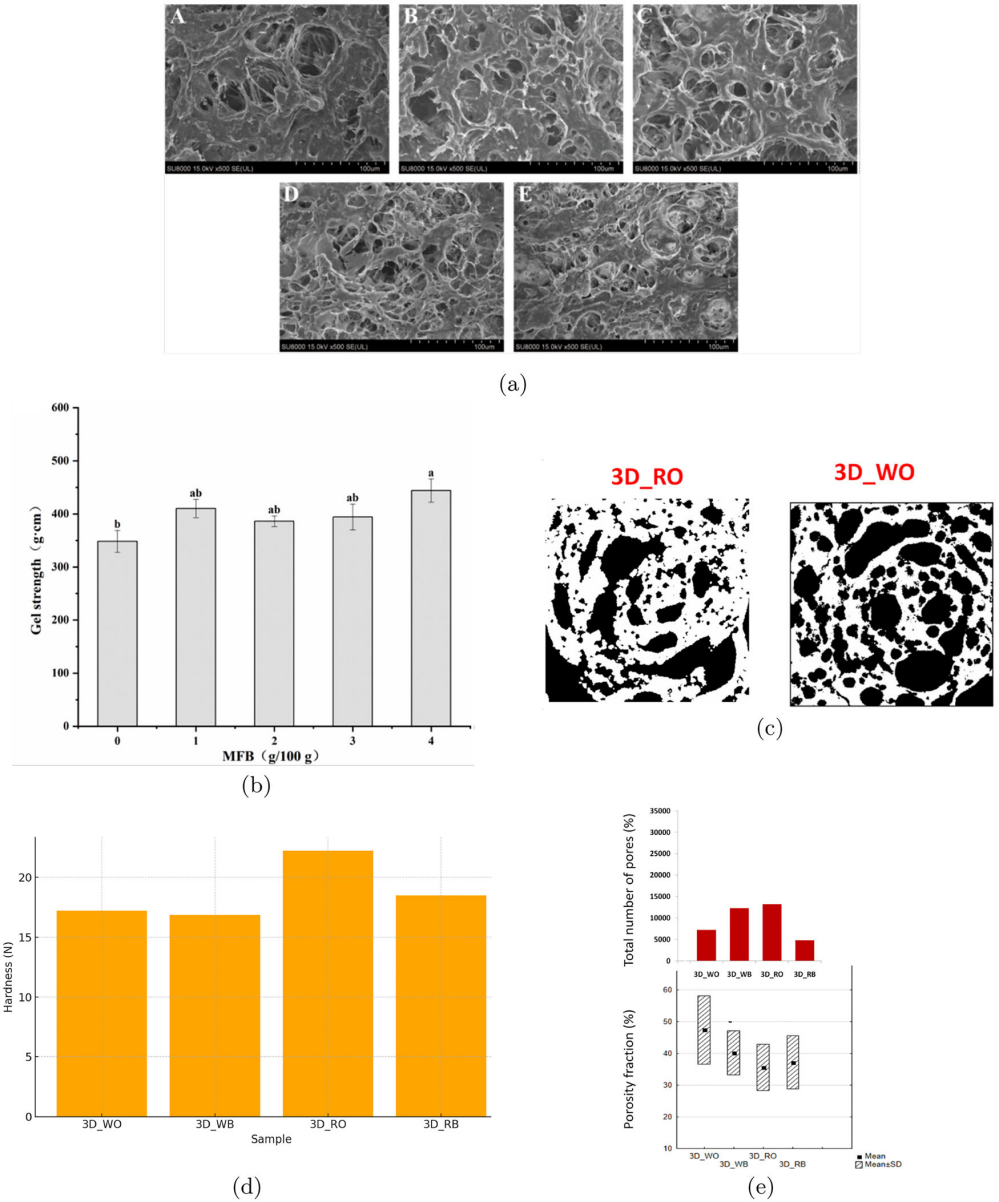
(a,b) Effects of MFB on the microstructure of the 3D printed samples (500 ×; A–E: The microtopography of surimi gel samples supplemented with 0, 1.0, 2.0, 3.0 and 4.0 g/100 g MFB, respectively), and gel strength of 3D‐printed surimi gel samples. Different letters indicate significant differences (P < 0.05). Adapted from Wei et al. (2024) with permissions from Elsevier. (c) Cross sectional XCT images of samples obtained by 3D printing and traditional manufacturing process. (d,e) Hardness and porosity of different 3D printed samples, wheat‐oil (WO), wheat‐rice‐oil (RO), wheat‐butter (WB), wheat‐rice‐butter (RB). Adapted from Derossi et al. (2020a) with permissions from Elsevier.

Previous studies indicate that increasing the average pore size, even if accompanied by a decrease in the total number of pores, often leads to reduced mechanical strength (Wei et al. 2024; Derossi et al. 2020a). For instance, Wei et al. (2024) demonstrated that surimi gel samples with larger pores (Figure 8a) exhibited lower gel strength, as shown in Figure 8b. Similarly, Derossi et al. (2020a) investigated wheat‐flour‐based 3D‐printed samples and observed that larger pores tended to decrease hardness compared to smaller pores (Figure 8c–e).

Structures characterized by a higher density of smaller pores typically exhibit enhanced mechanical properties, primarily due to the formation of thicker cell walls under these conditions. These thicker walls provide increased stiffness and resistance to deformation (Liu et al. 2020c; Yun et al. 2022; Yu et al. 2022b). Additionally, increasing the number of pores generally reduces the bulk density of a sample (Hussain et al. 2022; Varghese et al. 2020). While lower density can negatively affect absolute strength, it may enhance specific mechanical properties (strength‐to‐weight ratio), allowing similar performance with less material (Lombardi et al. 2025b).

A general trend is the inverse relationship between pore size and number, with larger pores typically occurring in smaller numbers and vice versa. This highlights the importance of assessing the total void fraction, as mechanical performance is determined by a balance of size, number, and connectivity. Increasing porosity usually results in reduced hardness due to lower material continuity, but mechanical performance cannot be predicted from pore size or number alone‐it emerges from the complex interplay of all these parameters.

### 5.2 Functional Properties

Beyond mechanical strength, porous structure critically affects the functional properties of 3D‐printed foods. Pore architecture influences mouthfeel, texture, chewability, and water/oil retention, all of which contribute to consumer perception and product quality. Open, interconnected pores increase permeability, enabling rapid absorption or release of liquids, which can be beneficial in products designed for rapid flavor release, hydration, or aeration. In contrast, closed‐cell or partially connected pores provide better moisture barrier properties, helping retain crispness or firmness over time.

Porosity also plays a key role in controlling thermal behavior. Smaller pores with low connectivity reduce heat transfer, improving thermal insulation‐useful for products that must retain warmth. Larger, more open pores promote faster cooling and can improve drying efficiency during post‐processing. These characteristics are not only relevant to food texture and shelf life but can also be tailored for non‐food applications such as bioactive scaffolds, where pore interconnectivity facilitates nutrient transport and cell growth.

Furthermore, the sensory properties of foods—such as crunchiness, creaminess, or aeration—are strongly linked to the micro‐ and macro‐structure of the porous network. For example, finely controlled microporosity can produce smooth, melt‐in‐mouth textures, while larger macropores contribute to airy, crispy structures. By adjusting pore morphology through both formulation and process design, it is possible to create a broad spectrum of textural experiences.

### 5.3 Hierarchical Design Strategies

The most promising approaches to balancing mechanical and functional requirements involve hierarchical porous structures, where large‐scale inter‐strand macropores are combined with fine intra‐strand microporosity. Such multiscale architectures, inspired by natural foams and engineered scaffolds (Tammaro et al. 2022a; Li et al. 2019), can achieve high stiffness‐to‐weight ratios, enhanced compression resilience, and effective energy absorption, while using less material overall.

In these systems, macropores reduce weight and enable fluid or gas exchange, while micropores within the filament walls help distribute stresses and provide additional functionality such as moisture control or controlled release of active ingredients. By carefully tuning the scale, distribution, and connectivity of these pores, designers can target multiple performance objectives simultaneously. This combination of structural efficiency, functional versatility, and reduced resource use makes hierarchical design a valuable strategy for advancing both product quality and sustainability in 3D‐printed food manufacturing.

## 6 Effect of 3D Food Printer Type

**FIGURE 9:**
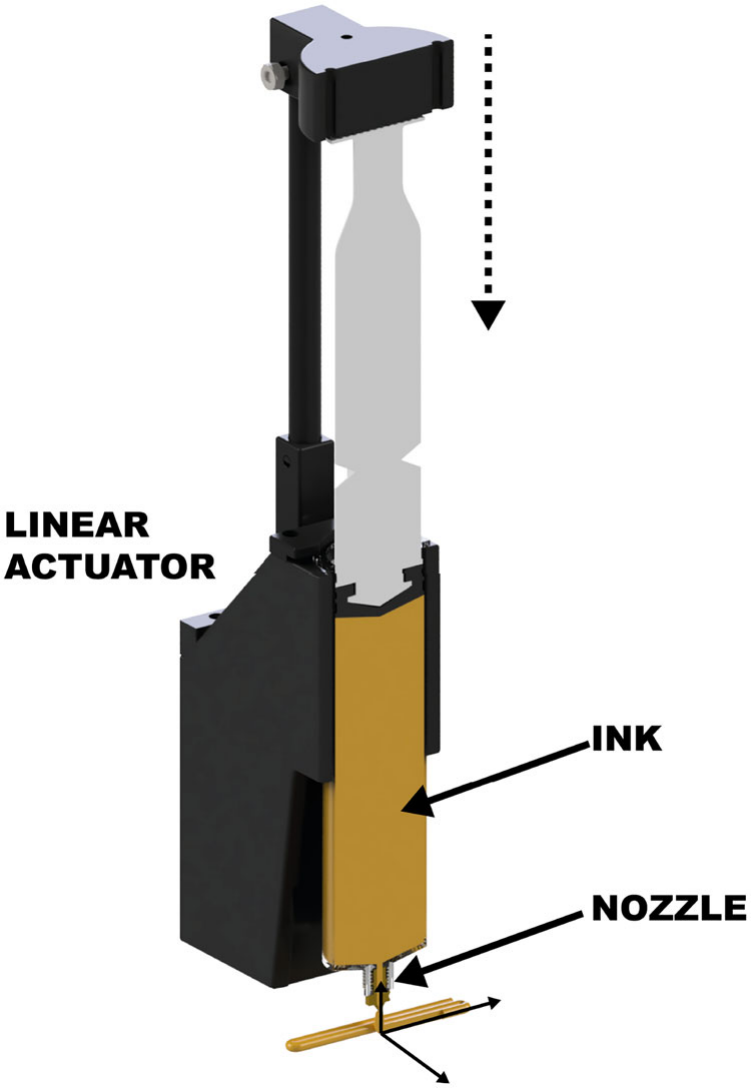
Schematic representation of a 3D food printing extrusion system, showing the linear actuator for vertical motion, the ink reservoir, and the nozzle for precise material deposition.

A typical 3D food printer operates on the principle of additive manufacturing, where food material is deposited layer by layer to create a three‐dimensional structure. The process begins with the preparation of a “food ink,” which is loaded into a cartridge or syringe. To extrude the ink through a nozzle, the printer uses either a mechanical system—such as a piston or screw mechanism (Liu et al. 2020c; Wen et al. 2022; Pant et al. 2021)—or a pneumatic system that relies on air pressure (Ahmadzadeh and Ubeyitogullari 2022, 2023a, 2023b; Bulut and Candoğan 2022) (see Figure 9).

In piston‐driven systems, the flow rate is directly controlled by the velocity of the piston's movement. In contrast, pneumatic systems operate based on an applied pressure drop, with the flow rate largely influenced by the nozzle geometry and the ink's viscosity. One potential drawback of pressure‐driven systems is their sensitivity to changes in the ink's rheological properties over time while it remains in the cartridge. Such changes can lead to fluctuations in the flow rate during printing, reducing precision and potentially compromising the quality of the final product.

Interestingly, the literature does not reveal a clear link between the type of printer and the resulting porous structure of 3D‐printed samples, such as pore size, cell structure type, or pore count. Moreover, there is a notable lack of systematic studies that isolate printer type as a variable while keeping ink composition and printing parameters constant. Conducting such research could yield valuable insights and play a critical role in advancing the design and optimization of 3D food printing systems.

## 7 Modeling

Modeling is useful for advancing 3D food printing by enabling the simulation and optimization of printing processes, material behavior, and the final properties of printed products. Among the most widely used techniques is computational fluid dynamics (CFD), which is employed to analyze the flow characteristics of food materials during extrusion. CFD helps optimize key processing parameters, such as layer height, deposition thickness, and nozzle geometry.

**FIGURE 10:**
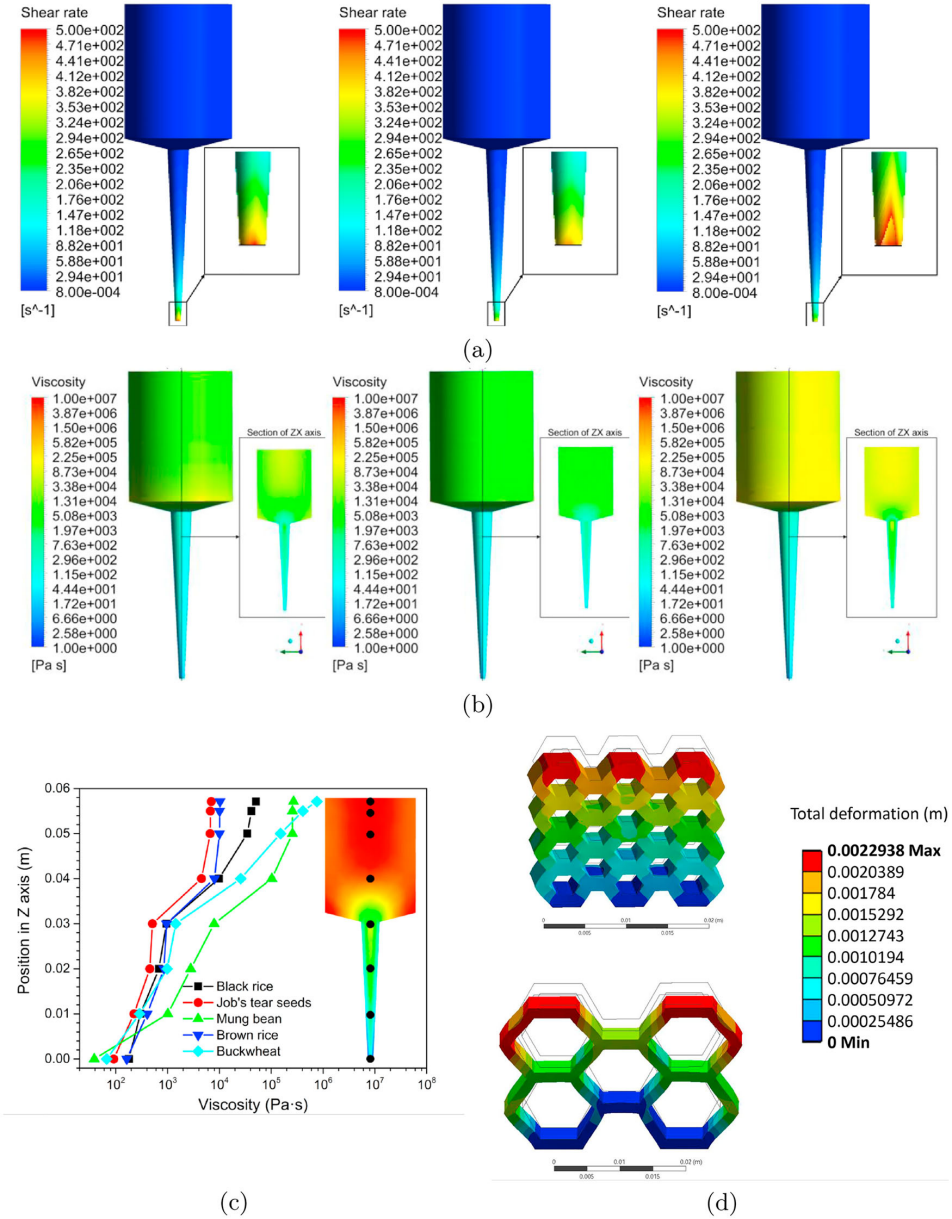
(a) Simulated local shear rate profiles and (b) viscosity profiles of different grain gels during printing. Data refer, from left to right, to black rice, job's tear seeds, mung bean, respectively. (c) Viscosity values taken at eight different positions along the syringe and nozzle centerline. Adapted from Guo et al. (2020) with permissions from Elsevier. (d) Compression simulations of the honeycomb structure having a cell size of 2 mm (top) and 5 mm (bottom) using FEM. Adapted from Vancauwenberghe et al. (2018) with permissions from Elsevier.

The majority of studies in the literature utilize CFD tools like ANSYS POLYFLOW and COMSOL Multiphysics to investigate the flow field within the nozzle during 3D food printing (Oyinloye and Yoon 2021; Guo et al. 2020; Yang et al. 2019; Guo et al. 2019; Singh and Muthukumarappan 2017, 2016; Woodfield et al. 2004; Azad Emin et al. 2011). Typical simulation results include the shear rate field within the syringe and nozzle (Figure 10a). Once a fluid constitutive equation is selected, the corresponding viscosity field can be calculated (Figure 10b,c), which also facilitates visualization of the fluid stress distribution. Less commonly, some researchers employ analytical models to estimate extrusion forces (Percoco et al. 2021) or predict the mechanical properties of printed structures (Lin et al. 2014; Kim et al. 2018).

Alternatively rheological parameters can be estimated by fitting models such as the Bird‐Carreau, Power‐Law, or Carreau‐Yasuda models to experimental rheological data (Yang et al. 2019). These parameters are used in CFD simulations to compute velocity field inside the nozzle, syringe, or just beyond the nozzle exit. From this velocity field, the shear rate distribution can be computed, which in turn allows for the calculation of the stress field.

An simple example for this is provided by Liu et al. (2020a) who showed that increasing plunger velocity results in higher shear rates, which can degrade print quality due to shear thinning. This effect, predicted by the rheological models, reflects a decrease in viscosity under high shear conditions. Other studies have investigated how different rheological properties influence flow behavior, offering valuable insights into material‐specific performance during 3D printing (Guo et al. 2020).

A key limitation of computational fluid dynamics (CFD) modeling in 3D food printing is its inability to capture the microstructural features of printed materials, as it treats food as a continuous fluid. This simplification overlooks the complex internal architecture of food structures. Additionally, the wide range of food materials used in 3D printing‐such as fruit juices, milk, gels, and meat pastes‐exhibit diverse rheological, thermodynamic, and physical properties. Since proper measurement of these properties is not always possible, CFD simulations may lack accuracy. Many of these materials also display viscoelastic and yield‐stress behaviors, which are often neglected in CFD models, potentially leading to inaccurate or incomplete predictions.

Beyond CFD, modeling efforts have also explored the mechanical properties of 3D‐printed samples. For example, researchers have applied both analytical modeling (Guessasma et al. 2011) and finite element method (FEM) simulations (Vancauwenberghe et al. 2018) to investigate the behavior of pectin‐based food structures with CAD‐designed macroporous architectures (Figure 10d). These models, based on assumptions of linear‐elastic deformation and small‐strain axial compression, showed excellent agreement with experimental data.

Other analytical approaches have gone further by incorporating finite elasticity and pseudo‐elasticity theories to better capture the nonlinear and inelastic responses of soft agricultural tissues under mechanical loading (Shirmohammadi et al. 2015). These methods offer a more nuanced understanding of how soft food materials behave under deformation, enhancing predictive capabilities.

In another example, Del Nobile et al. (Del Nobile et al. 2007) employed the generalized Maxwell model to investigate the viscoelastic properties of solid‐like foods. This approach effectively described stress relaxation behavior and provided valuable insights into the structural integrity and mechanical responses of various food matrices.

Similarly, Fahmy et al. (2023, 2022) explored how incorporating spherical cavities into 3D‐printed designs could create porous structures, demonstrating a clear relationship between compression hardness and porosity. However, the pores in these studies were design‐induced macropores on the millimeter scale, rather than intrinsic microstructural features.

Another promising direction involves the development of constitutive models that describe the large‐strain behavior of starch‐based 3D‐printed foods by linking the mechanical response of the porous architecture to the material's effective macroscopic properties (Jonkers et al. 2020). These models integrate experimental observations with numerical simulations to better understand how microstructure influences the overall mechanical performance of printed food products.

In summary, numerical modeling—whether through CFD, FEM, or analytical methods—remains a vital tool for understanding and optimizing 3D food printing. However, significant challenges remain, particularly in representing material diversity, viscoelastic behavior, and microstructural complexity. Addressing these gaps is essential to improve predictive accuracy and expand the utility of modeling techniques in the design of advanced food printing systems.

## 8 Conclusions and Prospects

### 8.1 Main Findings

This review has explored in detail the complex interplay between formulation strategies, printing parameters, and post‐processing treatments in shaping the porous microstructure of 3D‐printed food products. The main findings are summarized as follows:

- Formulation effect (Section 3): Increasing polysaccharide or protein concentrations generally leads to denser internal structures with smaller pores, thicker cell walls, and improved mechanical strength. High‐amylose starches favor fine pore formation through retrogradation, while protein‐rich inks enhance shape fidelity by promoting tighter molecular networks.
- Temperature effect (Section 4.1): Printing temperature is a critical parameter. Low temperatures often result in under‐gelatinized, loose structures, while moderate heating enhances gelatinization and denaturation, producing compact and homogeneous pores. Excessive temperatures, however, can degrade structural integrity and reduce porosity uniformity.
- Velocity effect (Sections 4.2 and 4.3): Printing and extrusion velocity impacts filament uniformity and porosity. Lower speeds tend to yield larger pores with consistent filament deposition, whereas higher speeds reduce porosity and promote compactness, although they may introduce shear‐induced defects.
- Nozzle diameter effect (Section 4.4): Nozzle size directly influences pore architecture. Smaller diameters yield tightly packed filaments and reduced porosity, resembling fibrous textures. Larger nozzles increase pore size and openness but reduce printing precision. Smaller nozzles also require higher extrusion pressures, potentially affecting print stability.
- Mechanical properties (Section 5): Pore architecture is closely tied to mechanical strength. Higher pore density with smaller pores improves resistance to deformation due to thicker wall formation. In contrast, larger pores reduce structural continuity and mechanical robustness, even if they reduce bulk density.

Despite these advances, current control over porosity in 3D food printing is largely dependent on post‐processing methods, particularly freeze‐drying. This limits scalability and energy efficiency. Controlling porous structure characteristics remains a complex challenge due to the inherent heterogeneity of these systems and the multitude of parameters involved in the process. Furthermore, the current state of research presents a fragmented landscape, with data often generated using diverse systems and lacking a systematic approach to parameter variation.

### 8.2 Future Prospects

To move toward more efficient and predictive control of porous structures in 3D‐printed food, future research should focus on the following directions:

1. Intrinsic control of porosity: Develop methods to tune porous structures directly through printing parameters such as temperature, flow rate, and velocity, eliminating the need for post‐processing.
2. Systematic parameter studies: Use simplified model formulations to isolate and study the effects of individual printing variables. This will facilitate clearer interpretation and improve reproducibility across studies.
3. Design of hierarchical porous structures: Explore the integration of multi‐scale porosity by combining inter‐strand macropores, defined by the printing path and spacing, with intra‐strand micropores, arising from material formulation and process conditions. This hierarchical approach could enable the creation of lightweight yet mechanically stable food structures with tailored textures and improved mouthfeel, while also offering opportunities to reduce material usage without compromising consumer satisfaction.
4. Rheology‐structure relationships: Establish direct links between rheological properties (e.g., viscoelasticity, shear‐thinning behavior) and pore formation. Understanding how rheology governs filament formation, air entrapment, and layer adhesion is key to structural control.
5. Advanced modeling and prediction: Improve existing computational and analytical models (e.g., CFD, FEM) to simulate pore formation during extrusion. Models should incorporate realistic, food‐specific rheological properties and account for non‐Newtonian behavior, phase separation, and structural transitions.

Addressing these challenges will enable more accurate, energy‐efficient, and tailored design of 3D‐printed foods‐advancing the field toward practical applications in digital gastronomy and personalized nutrition.

## Author Contributions

**Lorenzo Lombardi**: conceptualization, investigation, methodology, validation, formal analysis, data curation, writing – original draft, writing – review and editing. **Luigi Davide Gala**: data curation, writing – original draft, formal analysis.**Claudio Esposito**: data curation, formal analysis, writing – original draft.**Daniele Tammaro**: writing – original draft, writing – review and editing, supervision, formal analysis, project administration, funding acquisition, resources.

## Conflicts of Interest

The authors declare no conflicts of interest.
